# Current status and influencing factors of knowledge, attitude and practice of personal protection of healthcare workers in isolation wards of COVID-19 designated hospitals

**DOI:** 10.3389/fpubh.2025.1510015

**Published:** 2025-05-14

**Authors:** Qian Liu, Yan Li, Chao Han, Qi Li

**Affiliations:** ^1^Shandong Public Health Clinical Center, Shandong University, Jinan, Shandong, China; ^2^Department of Psychiatry, Shandong Mental Health Center, Shandong University, Jinan, Shandong, China

**Keywords:** COVID-19, HCW, personal protection, KAP, mental state

## Abstract

**Objectives:**

The isolation treatment and special care of COVID-19 patients expose frontline healthcare workers (HCWs) involved in treatment to more risks of infection exposure. Therefore, investigating the current status of personal protection KAP of HCWs in isolation wards and its influencing factors will be helpful in improving personal protection in major public health events.

**Methods:**

Research data came from COVID-19 designated medical institutions in Shandong Province from October to December 2022, and were collected through questionnaire surveys. The questionnaire is self-designed and composed of general information questionnaire, KAP questionnaire and anxiety and depression questionnaire. Univariate factor analysis and multiple linear regression analysis were used to study the influencing factors of KAP of the research subjects.

**Results:**

In terms of KAP Scores, the mean knowledge score was 6.82 ± 1.6, with 85.60% scoring at a medium level or below. The attitude mean score was 27.56 ± 4.1, and 78.60% held a favorable disposition toward personal protection measures. For practice, the mean score was 44.44 ± 5.6, with approximately 30.86% demonstrating room for improvement. Univariate analysis indicated significant differences in knowledge scores among HCWs with varying numbers of personal protective equipment (PPE) trainings and levels of depression and anxiety (*p* ≤ 0.05). Attitude scores differed significantly based on depression and anxiety levels (*p* ≤ 0.05). Practice scores varied significantly according to occupation, work experience, cumulative working time in isolation wards, and PPE training frequency (*p* ≤ 0.05). Multiple linear regression analysis revealed that increased PPE training frequency (*β* = 0.168, *p* = 0.007) and lower anxiety levels (*β* = −0.256, *p* ≤ 0.001) were associated with higher knowledge scores. Depression levels (*β* = −0.208, *p* = 0.001) were negatively associated with attitudes. Longer medical experience (*β* = 0.132, *p* = 0.029), more cumulative working time in isolation wards (*β* = 0.310, *p* ≤ 0.001), and lower anxiety levels (*β* = −0.129, *p* = 0.034) positively influenced practice scores.

**Conclusion:**

In summary, for HCWs in isolation wards, increasing the frequency of protective training and reducing anxiety will improve their personal protection knowledge; alleviating depression will cultivate a positive attitude toward personal protection; and relieving anxiety, along with longer job tenure and working hours, were associated with enhanced protective behaviors.

## Introduction

The emergence of COVID-19 in late 2019 has presented a significant challenge to the global healthcare system. The pandemic’s case fatality rate is estimated at approximately 1–3% (1), surpassing that of SARS and general influenza viruses (2). Unprecedentedly, stringent social distancing measures have been universally implemented worldwide to combat the outbreak. In response to this unprecedented crisis, numerous countries have established specialized hospitals dedicated to providing comprehensive care for patients with COVID-19. These medical facilities incorporate designated isolation wards aimed at curtailing viral transmission and safeguarding healthcare professionals from potential infections (3).

The COVID-19 pandemic has significantly increased the challenges and risks faced by frontline healthcare workers (HCWs), and it is imperative to implement effective personal protective measures in COVID-19 isolation wards to reduce nosocomial infection. Investigations have shown that the relative risk of developing COVID-19 is related to factors such as PPE, occupation, and exposure (4). Globally, studies from European settings have corroborated similar challenges. A German study by Müller et al. (5) found 68% of dentists faced difficulties implementing COVID-19 protocols, highlighting universal training needs (5). Furthermore, Polish research by Malczynska et al. (6) revealed 42% HCWs developed PPE-related dermatoses, demonstrating cross-regional occupational health impacts. The KAP model, encompassing Knowledge (understanding of disease transmission mechanisms/prophylactic protocols), Attitude (willingness to adopt and improve preventive behaviors), and Practice (consistent adherence to infection control measures), due to its feasibility and effectiveness, has been widely applied in nosocomial infection control research (7–9). Previous studies have demonstrated that inadequate knowledge and negative attitudes toward PPE protocols are associated with suboptimal adherence in high-risk clinical settings (10). However, there is still a lack of research on the KAP of HCWs, especially those working in the COVID-19 isolation wards.

HCWs faced challenges in PPE compliance, with only 54.9% consistently using PPE in the Democratic Republic of the Congo (11) and 13.8% correctly donning PPE in Saudi Arabia (12). These gaps reflect KAP disconnects adequate knowledge but poor attitudes and practices, demonstrating incomplete translation of knowledge into action. Given the unique nature of COVID-19 and the demands on specialized care, it is critical to understand the current status of personal protection KAP among HCWs in isolation wards of designated hospitals for COVID-19, focusing on the HCWs directly involved in patient care. This study investigated these aspects to determine the factors that influence HCWs’ adherence to personal protective measures. The findings of this study will help develop effective strategies to promote personal protective measures in these settings, ultimately improving HCWs’ safety and helping prevent the spread of COVID-19.

## Methods

### Research participants

The study employed a cross-sectional design, recruiting a sample of 243 HCWs from the isolation ward of a designated hospital for COVID-19 treatment in Shandong Province between October and December 2022, including doctors, both registered and assistant nurses, and other medical professionals from supporting departments (laboratory, radiology and infection control specialists). Convenience sampling method was utilized to enroll participants who met the inclusion criteria, which encompassed (1) medical and related personnel engaged in close-loop work within isolation wards, and (2) voluntary willingness to participate in the study. Exclusion criteria were applied to staff not subjected to closed-loop management (i.e., personnel not residing in designated accommodations or permitted unrestricted movement outside isolation wards).

### Research measures

(1) General information questionnaire: designed by the researchers, including various potential influencing factors on KAP, such as gender, age, professional title, working years, type of work, marital status, children’s status, frequency of hospital infection prevention training, etc. (Appendix 2). (2) KAP questionnaire: based on the theory of KAP, referring to relevant literature at home and abroad, and referring to the “Prevention and Control Plan of COVID-19 Disease (Third Edition)” and other relative national standards, combined with the discussion of the research group, and then through expert consultation and pre-survey, the formal questionnaire was gradually modified (Appendix 3). (3) Anxiety and depression questionnaire: the Chinese version of Beck Anxiety Inventory (BAI) and Beck Depression Inventory (BDI) were used to evaluate the mental health status of the HCWs in the isolation ward (Appendix 4). The design and reporting of this study followed the STROBE (Strengthening the Reporting of Observational Studies in Epidemiology) guidelines.

The knowledge dimension consisted of 10 multiple choice questions, encompassing the fundamental principles of personal protection, intricate details regarding protective clothing, and the selection process for protective equipment. A correct response was awarded 1 point, while an incorrect answer received 0 points, resulting in a maximum score of 10. The attitude dimension comprised 6 multiple choice questions that gauged individuals’ attitudes toward personal protection knowledge and skills, their perspective on training programs, as well as their commitment to implementing personal protection measures. The Likert scale with a range of 1 to 5 points was employed to assess responses from “strongly disagree” to “strongly agree,” yielding an overall score between 6 and 30. A higher score indicated a more positive attitude. The practice dimension included 10 multiple choice questions primarily focusing on active learning and practical application of personal protection-related knowledge and skills. The Likert scale with values ranging from 1 to 5 assigned scores corresponding to “never,” “occasionally,” “sometimes,” “often,” and “always” respectively. The total score ranged from 10 to 50, with a higher score indicating better adherence to desired behaviors. The standard score for each dimension is calculated by dividing the average score by the full score and multiplying it by 100. An excellent standard score is above 85, a moderate score ranges from 60 to 85, and a poor score is below 60.

Depressive status was assessed using the BDI. According to the rating criteria of the scale, scores ranging from 0 to 4 were categorized as absence of depression, scores between 5 and 13 indicated mild depression, scores between 14 and 20 denoted moderate depression, while scores ≥ 21 represented severe depression. Anxiety status was evaluated using the BAI, based on the assessment guidelines of this scale, a score of ≥45 indicated anxiety, whereas a score below <45 indicated no presence of anxiety.

The questionnaire content was evaluated by seven specialists in hospital infection management, resulting in a content validity index of 0.946. To ensure the reliability and validity of the questionnaire, a pre-survey was conducted with 20 isolation ward HCWs who met the inclusion and exclusion criteria. The Cronbach’s *α* coefficients for knowledge, attitude, and practice dimensions were found to be 0.749, 0.942, and 0.862 respectively; yielding a total Cronbach’s *α* coefficient of 0.894 for the questionnaire along with a retest reliability coefficient of 0.798. Previous studies have demonstrated good reliability and validity for Chinese versions of BAI and BDI (13, 14), thus further calculations were deemed unnecessary.

### Data collection

Data collection was conducted using an online tool called ‘Wenjuanxing’ to create the questionnaire and generate a two-dimensional code image for distribution. The principal investigator of this study communicated with the department head and head nurse of the selected hospital to clarify the target population, research objectives, survey methodology, and informed consent considerations. Strict adherence to scientific research rules was maintained throughout. The questionnaire was administered anonymously with informed consent from participants, and a one-week timeframe was agreed upon for data retrieval. All questions were designated as mandatory, allowing only one response per IP address. A total of 300 questionnaires were distributed, resulting in 271 responses. After excluding incorrect or invalid responses, 243 valid questionnaires were obtained, yielding an effective response rate of 89.67%. The high response rate can be attributed to two factors: firstly, the occupational health survey was required in the closed-loop management policy at that time. Secondly, mobile based questionnaires allowed for real-time completion during work breaks, minimizing non-response bias to the greatest extent possible.

### Statistical analysis

The outcome variables of this study included the knowledge dimension, assessed through a 10-item multiple-choice questionnaire; the attitude dimension, evaluated using a 6-item Likert scale questionnaire; and the practice dimension, measured with a 10-item Likert scale questionnaire. Exposure variables were the frequency of PPE training, years of work experience, cumulative working time in isolation wards, and levels of depression and anxiety. Potential confounders such as gender, age, profession, professional title, marital status, and parental status were considered in the univariate analysis. Multiple linear regression analysis was used to control for these confounders, focusing on the significant variables identified in the univariate analysis.

The data was imported into Excel and a database was established, which underwent a rigorous accuracy check by two individuals. Analysis was conducted using SPSS 22.0 software. Count data were described in terms of frequency and percentage (%), while normally distributed measurement data were expressed as mean ± standard deviation. Group comparisons were performed using either the two independent samples *t*-test or one-way analysis of variance. Multiple linear regression was employed to analyze the factors influencing personal protection KAP among HCWs in isolation wards of designated hospitals. Statistical significance was determined at *p* ≤ 0.05.

## Results

### Demographic characteristics of participants

A total of 243 HCWs from COVID-19 isolation wards participated in this study. Key demographic and occupational characteristics are summarized in Table 1. The cohort predominantly comprised nurses (62.1%) and doctors (32.1%), with 41.2% having over 10 years of medical experience. Most participants (60.9%) attended 1–2 PPE training sessions, while 13.2% received more than 5 trainings. Psychological assessments revealed that 32.9% experienced anxiety, and 28% reported varying degrees of depression (10.3% severe). Factors significantly linked to KAP outcomes, including work experience, cumulative days in isolation wards, training frequency, and mental health status, are analyzed in subsequent sections. For additional details (e.g., age distribution, marital status, parental status), refer to Table 1.

**Table 1:** Demographic, occupational, and psychological characteristics of participants (*n* = 243).

Variable	Statistics *n* (%)
Gender	Female: 183 (75.3); Male: 60 (24.7)
Age	18–25 years: 59 (24.3); 26–30 years: 54 (22.2); 31–40 years: 96 (39.5); 41–50 years: 28 (11.5); 51–60 years: 6 (2.5)
Occupation	Doctor: 78 (32.1); Nurse: 151 (62.1); Other positions: 14 (5.8)
Professional title	None/Assistant: 4 (1.6); Junior: 126 (51.9);Intermediate: 83 (34.2); Senior: 30 (12.3)
Work experience in medical industry	<1 year: 33 (13.6); 1–3 years: 35 (14.4); 4–6 years: 28 (11.5); 7–10 years: 47 (19.3); > 10 years: 100 (41.2)
Marital status	Married: 144 (59.3); Single: 99 (40.7)
Parental status	No children: 114 (46.9); Adult children: 9 (3.7); Minor children with support: 68 (28.0); Minor children without support: 52 (21.4)
Isolation ward work days	0–14 days: 77 (31.7); 15–30 days: 41 (16.9); 31–60 days: 82 (33.7); 61–100 days: 24 (9.9); >100 days: 19 (7.8)
PPE training sessions	None: 11 (4.5); 1–2 times: 148 (60.9); 3–5 times: 52 (21.4); >5 times: 32 (13.2)
Depression level	None: 175 (72.0); Mild: 24 (9.9); Moderate: 19 (7.8); Severe: 25 (10.3)
Anxiety status	No anxiety: 163 (67.1); Anxiety: 80 (32.9)

### The current status of personal protection KAP and psychological state

In the study, the knowledge dimension included 10 multiple-choice questions (0–10 points: 1 = correct, 0 = incorrect). The attitude dimension used a 5-point Likert scale (1 = strongly disagree to 5 = strongly agree), totaling 6–30 points. The practice dimension also employed a 5-point Likert scale (1 = never to 5 = always), with a range of 10–50 points. In the study, it was observed that the mean score for the dimension of personal protection knowledge among 243 workers was 6.82 ± 1.6, with a range from 2 to 10. Among them, a majority of 208 workers (85.60%) exhibited a moderate or lower level of knowledge in this domain. The mean score for the dimension of personal protection attitude was found to be 27.56 ± 4.1, ranging from 6 to 30, with approximately 52 individuals (21.40%) demonstrating a moderate or lower level of attitude toward personal protection practice. Furthermore, the mean score for the dimension of personal protection practice was determined as 44.44 ± 5.6, ranging from 20 to 50; within this group, around 75 participants (30.86%) displayed a moderate or lower level of adherence to recommended protective behaviors. The specific scores of each item are shown in Tables 2–4.

**Table 2:** The scores for specific items in knowledge dimension.

Questions	Minimum value	Maximum value	Scores (Mean ± SD)
K1: According to the “Diagnosis and Treatment Plan of COVID-19 Disease (Ninth Edition),” what are the main transmission routes of COVID-19 virus?	0	1	0.38 ± 0.49
K2: What is the incubation period after infection with Omicron variants?	0	1	0.36 ± 0.48
K3: What should staff in the isolation ward wear when entering the contaminated area?	0	1	0.64 ± 0.48
K4: What should staff in the isolation ward do when removing their protective masks?	0	1	0.94 ± 0.24
K5: Which statement about hand hygiene is correct?	0	1	0.55 ± 0.49
K6: When wearing a protective mask, a tightness test should be performed on the protective mask. Which of the following statements is correct?	0	1	0.77 ± 0.42
K7: When removing protective equipment in the first removal area, which of the following statements is incorrect?	0	1	0.93 ± 0.26
K8: When removing protective equipment in the second removal area, which of the following statements is incorrect?	0	1	0.82 ± 0.39
K9: When in a contaminated area, regarding what should be done if protective clothing is damaged, which of the following statements is correct?	0	1	0.49 ± 0.5
K10: In a contaminated area, regarding what to do if a protective mask falls off or becomes loose, which of the following statements is correct?	0	1	0.91 ± 0.29

K1–K10 represent 10 questions in the knowledge dimension (see Appendix 3 for details).

Scoring criteria: 1 point for correct answers, 0 for incorrect answers.

**Table 3:** The scores for specific items in attitude dimension.

Questions	Minimum value	Maximum value	Scores (mean ± SD)
A1: Do you think it is important to conduct training and assessment on hospital infection knowledge and putting on and taking off protective equipment?	1	5	4.69 ± 0.72
A2: Do you think it is important to be familiar with the brands and models of protective equipment commonly used in our hospital?	1	5	4.36 ± 0.99
A3: Do you think it is important to master necessary occupational protection knowledge to reduce occupational exposure hazards caused by COVID-19?	1	5	4.72 ± 0.70
A4: Do you think it is necessary to learn emergency response procedures for occupational exposure in isolation wards?	1	5	4.68 ± 0.75
A5: Do you think that repeatedly practicing putting on and taking off protective equipment and watching videos showing the details of putting on and taking off protective equipment will help you improve your skills and prevent occupational exposure?	1	5	4.63 ± 0.79
A6: How effective do you think existing protective equipment is in protecting medical staff?	1	5	4.48 ± 0.85

A1–A6 represent 6 questions in the attitude dimension (see Appendix 3 for details).

5-point Likert scale: 1 = strongly disagree, 5 = strongly agree.

**Table 4:** The scores for specific items in practice dimension.

Questions	Minimum value	Maximum value	Scores (mean ± SD)
P1: Have you taken the initiative to repeatedly watch the video showing the process of putting on and taking off protective equipment and inquire about relevant prevention and control knowledge?	1	5	4.12 ± 0.94
P2: Have you actively checked and studied COVID-19 prevention and control plans issued by the country?	1	5	3.98 ± 1.03
P3: When taking off protective equipment, if you are not sure whether there is contamination, do you take the initiative to review the surveillance video to find out the reason?	1	5	3.71 ± 1.25
P4: Have you taken the initiative to ask experienced colleagues, head nurses, and full-time hospital infection control personnel for advice on protective equipment?	1	5	4.09 ± 0.98
P5: In the removal area, do you perform hand hygiene as required every time?	2	5	4.82 ± 0.50
P6: Are you able to strictly implement the COVID-19 hospital infection prevention and control regulations in the isolation ward?	2	5	4.76 ± 0.56
P7: When you take off your protective clothing, can you avoid contaminating the inner scrubs?	2	5	4.71 ± 0.58
P8: In the removal area, do you perform hand hygiene for a sufficient length of time each time?	2	5	4.74 ± 0.53
P9: When you wear a protective mask, do you perform an effective tightness test every time?	2	5	4.82 ± 0.46
P10: When you take off your protective mask, can the vibration of the mask be controlled to a small range?	1	5	4.70 ± 0.63

P1–P10 represent 10 questions in the practice dimension (see Appendix 3 for details).

5-point Likert scale: 1 = never, 5 = always.

### Univariate factor analysis of influencing factors of personal protection KAP

The results of the univariate analysis indicated significant differences in knowledge dimension scores among HCWs with varying numbers of protective equipment trainings and degrees of depression and anxiety (*p* ≤ 0.05). Attitude dimension scores also showed significant differences among HCWs with different levels of depression and anxiety (*p* ≤ 0.05). Furthermore, practice dimension scores exhibited significant differences among HCWs with diverse occupations, years of work experience, cumulative working time in isolation wards, and number of protective equipment trainings received (*p* ≤ 0.05). The specific analysis results are shown in Table 5.

**Table 5:** Univariate factor analysis of influencing factors of KAP of personal protection.

Items	Number of people	Knowledge dimension			Attitude dimension			Practice dimension		
		Scores (mean ± SD)	*t/F* value	*p-*value	Scores (mean ± SD)	*t/F* value	*P-*value	Scores (mean ± SD)	*t/F* value	*P*-value
Gender										
Male	60	44.68 ± 5.69	0.39	0.696	27.02 ± 4.99	−1.17	0.243	6.55 ± 1.78	−1.46	0.145
Female	183	44.36 ± 5.63			27.73 ± 3.74			6.90 ± 1.56		
Age (years old)										
18–25	59	44.46 ± 6.67	0.34	0.850	27.88 ± 3.87	0.94	0.440	6.69 ± 1.70	2.19	0.070
26–30	54	43.72 ± 6.11			27.94 ± 3.38			6.41 ± 1.50		
31–40	96	44.82 ± 4.88			26.98 ± 4.92			7.17 ± 1.55		
41–50	28	44.32 ± 4.60			27.71 ± 2.64			6.71 ± 1.72		
51–60	6	45.00 ± 7.04			29.17 ± 0.98			6.50 ± 1.87		
Occupation										
Doctor	78	44.92 ± 5.55	0.55	0.580	27.05 ± 5.13	1.15	0.320	6.68 ± 1.56	3.25	0.040
Nurse	151	44.14 ± 5.80			27.86 ± 3.09			6.79 ± 1.60		
Others	14	44.93 ± 4.29			27.00 ± 6.41			7.86 ± 1.92		
Professional title										
None or assistant title	4	46.75 ± 4.27	0.90	0.440	30.00 ± 0.00	1.62	0.180	7.50 ± 1.91	1.89	0.130
Junior title	126	44.30 ± 6.24			27.71 ± 4.02			6.60 ± 1.66		
Intermediate title	83	44.06 ± 5.14			26.90 ± 4.62			7.11 ± 1.51		
Senior title	30	45.73 ± 4.20			28.33 ± 2.52			6.80 ± 1.63		
Working experience in medical industry (years)										
<1	33	43.70 ± 7.78	1.20	0.310	27.45 ± 4.65	0.49	0.740	6.09 ± 1.67	2.52	0.042
1–3	35	44.11 ± 6.20			27.23 ± 4.94			6.71 ± 1.53		
4–6	28	45.46 ± 5.24			28.32 ± 2.88			6.75 ± 1.78		
7–10	47	43.28 ± 5.18			27.09 ± 4.68			7.19 ± 1.47		
>10	100	45.05 ± 4.84			27.70 ± 3.55			6.93 ± 1.61		
Marital status										
Married	144	44.42 ± 5.29	−0.04	0.970	27.26 ± 4.48	−1.36	0.180	6.90 ± 1.56	1.02	0.310
Single	99	44.45 ± 6.14			27.98 ± 3.40			6.69 ± 1.71		
Children’s status										
No children	114	44.47 ± 6.28	0.95	0.420	28.04 ± 3.38	2.38	0.071	6.67 ± 1.67	2.00	0.120
Adult children (over 16 years old)	9	45.67 ± 6.61			28.44 ± 3.61			5.89 ± 2.15		
Minor children (less than 16 years old, with a capable second guardian to assist)	68	45.00 ± 4.18			27.59 ± 4.74			7.03 ± 1.58		
Minor children (less than 16 years old, without a capable second guardian to assist)	52	43.40 ± 5.64			26.29 ± 4.48			7.02 ± 1.39		
Cumulative number of days working in the isolation ward (days)										
0–14	77	44.60 ± 6.40	0.67	0.620	28.00 ± 2.99	1.00	0.410	6.14 ± 1.70	7.05	<0.001
15–30	41	43.27 ± 6.50			27.51 ± 4.11			6.80 ± 1.33		
31–60	82	44.79 ± 4.78			27.66 ± 3.81			7.02 ± 1.48		
61–100	24	44.04 ± 4.78			27.00 ± 5.49			7.54 ± 1.53		
>100	19	45.26 ± 4.83			26.05 ± 6.42			7.74 ± 1.59		
Number of times participating in protective equipment training										
None	11	40.45 ± 8.70	3.73	0.012	26.36 ± 4.18	0.70	0.550	6.45 ± 1.75	5.36	0.001
1–2	148	44.14 ± 5.78			27.48 ± 3.97			6.53 ± 1.51		
3–5	52	44.81 ± 4.74			28.13 ± 2.79			7.31 ± 1.67		
>5	32	46.59 ± 4.11			27.34 ± 5.99			7.47 ± 1.65		
Depression level										
No depression	175	45.49 ± 4.68	9.22	<0.001	28.14 ± 3.66	5.93	<0.001	6.82 ± 1.62	1.63	0.180
Mild depression	24	40.08 ± 6.55			25.63 ± 5.09			7.29 ± 1.68		
Moderate depression	19	42.16 ± 6.34			27.53 ± 2.97			6.89 ± 1.41		
Severe depression	25	43.00 ± 7.57			25.28 ± 5.29			6.28 ± 1.67		
Anxiety level										
No anxiety	163	45.50 ± 4.47	4.73	<0.001	27.98 ± 4.01	2.73	0.019	6.95 ± 1.59	1.88	0.061
Anxiety	80	42.26 ± 7.01			26.67 ± 4.11			6.54 ± 1.65		

*U*/*F* values: *t*-values for independent *t*-tests, *F*-values for ANOVA.

*P* ≤ 0.05 indicates statistically significant differences between groups.

### Multiple linear regression analysis of influencing factors on personal protection KAP

Multiple linear regression analysis was conducted using the KAP scores of HCWs in isolation wards as the independent variable. The independent variables for non-linear regression analysis were selected based on statistically significant differences observed in univariate analysis (*α*_in_ = 0.05, *α*_out_ = 0.10). The specific assignment method is described in detail in Table 6.

**Table 6:** Multiple linear regression analysis independent variable assignment method.

Independent variable	Assignment method
Occupation	Doctor = 1; Nurse = 2; Others = 3
Working experience in medical industry	<1 year = 1; 1–3 years = 2; 4–6 years = 3; 7–10 years = 4; >10 years = 5
Cumulative number of days working in the isolation ward	0–14 days = 1; 15–30 days = 2; 31–60 days = 3; 61–100 days = 4; >100 days = 5
Number of times attended protective equipment training	0 time = 1; 1–2 times = 2; 3–5 times = 3; >5 times = 4
Depression level	No depression = 1; Mild depression = 2; Moderate depression = 3; Severe depression = 4;
Anxiety level	No anxiety = 1; Anxiety = 2

The multiple linear regression analysis identified statistically significant associations between predictors and KAP dimensions. For the knowledge dimension, each additional PPE training session was associated with a 1.222-point increase in knowledge scores (*β* = 0.168, *p* = 0.007). Higher anxiety levels showed a negative correlation with knowledge scores, with each unit increase in anxiety reducing scores by 3.068 points (*β* = −0.256, *p* < 0.001). In the attitude dimension, greater depression severity was linked to lower attitude scores, with each incremental increase in depression severity associated with a 1.08-point decrease (*β* = −0.208, *p* = 0.001). Regarding practice dimension, longer medical work experience correlated with higher practice scores (0.145-point increase per year, *β* = 0.132, *p* = 0.029), while cumulative days working in isolation wards had the strongest positive impact (0.403-point increase per day, *β* = 0.310, *p* < 0.001). Higher anxiety levels also negatively affected practice scores (0.442-point decrease, *β* = −0.129, *p* = 0.034). These results indicate independent associations between training frequency, psychological factors, and work-related experience with distinct dimensions of HCWs’ personal protection competencies. The data is shown in Table 7.

**Table 7:** Multiple linear regression analysis of influencing factors on personal protection KAP.

Variable	Partial regression coefficient (B)	Standard error	Standardized regression coefficient (β)	*t* value	*P*-value
Knowledge dimension^a^					
Constant term	45.542	1.560	–	29.200	<0.001
Number of times attended protective equipment training	1.222	0.446	0.168	2.739	0.007
Anxiety level	−3.068	0.735	−0.256	−4.174	<0.001
Attitude dimension^b^					
Constant term	29.134	0.544	–	53.524	<0.001
Depression level	−1.083	0.329	−0.208	−3.298	0.001
Practice dimension^c^					
Constant term	5.892	0.408	–	14.437	<0.001
Working experience in medical industry	0.145	0.066	0.132	2.190	0.029
Cumulative number of days working in the isolation ward	0.403	0.078	0.310	5.133	<0.001
Anxiety level	−0.442	0.207	−0.129	−2.138	0.034

^a^*R*^2^ = 0.101; Δ*R*^2^ = 0.028; *F* = 7.501; *P* = 0.007; ^b^*R*^2^ = 0.043; Δ*R*^2^ = 0.043; *F* = 10.875; *P* = 0.001; ^c^*R*^2^ = 0.136; Δ*R*^2^ = 0.017; *F* = 4.571; *P* = 0.034; “–” indicates no relevant data.

## Discussion

This study aimed to evaluate the KAP related to personal protection among HCWs in the isolation wards of a designated COVID-19 treatment hospital. Employing a cross-sectional design, we surveyed 243 medical personnel using a structured questionnaire to assess KAP and mental health statuses through the BAI and BDI. The principal findings revealed that HCWs generally demonstrated positive attitudes and practices toward personal protection, although knowledge deficiencies were identified. Higher frequency of PPE training and lower anxiety levels were associated with improved personal protection knowledge, while lower depression levels were associated with a more positive attitude. HCWs with greater work experience, longer tenure in the isolation area, and lower anxiety levels showed a higher likelihood of adhering to personal protection practices.

### Comprehensive analysis of KAP regarding personal protection among HCWs in isolation wards

The results of this survey provide a comprehensive analysis of the current status of KAP regarding personal protection among HCWs in isolation wards of designated hospitals. Our findings reveal that insufficient personal protection knowledge and skills, coupled with weak protection awareness, often lead to increased hazards in the prevention and control of infectious diseases. Specifically, the knowledge dimension score pertaining to personal protection is alarmingly low, with an average of 6.82 ± 1.6 points out of a possible 10. A staggering 85.60% of the HCWs (208 individuals) scored at the medium level or below, indicating a subpar understanding of personal protection measures. While this finding aligns with concerns raised by Müller et al. (5) regarding the importance of knowledge in infection control, our cross-sectional study design does not permit direct causal inferences between knowledge deficits and increased infection hazards. Further longitudinal or intervention studies are needed to elucidate the relationship between knowledge levels and actual infection outcomes. Nevertheless, the observed knowledge gaps highlight the urgent need for targeted educational interventions to optimize personal protection practices in high-risk clinical settings. Our observations further show that HCWs exhibit limited proficiency in fundamental theoretical knowledge related to the novel coronavirus (Table 2, K1, K2) and protocols for decontamination in the contaminated area (Table 2, K9). This could be attributed to their exposure to high psychological stress levels and demanding shift work schedules, which limit their time and energy for acquiring and staying updated with relevant professional knowledge (15). Given the ongoing evolution of the COVID-19 virus at that time and the need for constant updates and adjustments in prevention and control strategies, it is imperative for HCWs to receive enhanced theoretical training focusing on key aspects. In contrast, a study conducted in Saudi Arabia found that healthcare professionals received adequate training, which served as a foundational requirement for effective risk management and infection control policies (16).

Despite these deficiencies in knowledge, it is encouraging to note that the majority of HCWs in isolation wards exhibited a positive attitude toward personal protection, with an average score of 27.56 ± 4.1. Over 78.60% of the HCWs demonstrated a favorable disposition toward personal protection measures. This finding resonates with studies across diverse settings. Kim et al. (17) found that 81% of nurses in South Korea viewed PPE as essential for infection control, despite challenges in consistent adherence. Similarly, a multinational survey by Nwagbara et al. (8) noted that 76% of African HCWs expressed strong confidence in PPE efficacy, underscoring a global trend of positive attitudes amidst knowledge-practice gaps. Additionally, hospital leaders and colleagues may have influenced the formation of a culture that values personal protection. A positive outlook on personal protection can enhance motivation and confidence in implementing protective measures, thereby improving overall safety (18). Therefore, it is recommended to continue providing psychological support and encouragement to isolation ward working HCWs to boost their self-efficacy and confidence in personal protection.

At the level of personal protective practice, the research findings indicate that HCWs’ adherence to safety protocols is above average, with an average score of 44.44 ± 5.6. However, there is still room for improvement, particularly among a subset of HCW members who failed to actively seek information and familiarize themselves with newly issued national guides for COVID-19 prevention and control (Table 4, P2). Additionally, some HCWs neglected reviewing surveillance footage to identify potential sources of contamination when uncertain during PPE removal (Table 4, P3). These issues may reflect deficiencies in commitment toward enhancing personal protection practices. Conversely, HCWs demonstrated commendable efforts in implementing more explicit requirements outlined in the guidelines, such as hand hygiene within the removal area (Table 4, P5) which suggests high compliance with clearly specified requirements. The limitations in knowledge and skills, coupled with constraints imposed by the working environment and conditions, such as the availability, quality, and comfort of PPE, may impact the efficiency and effectiveness of implementing personal protective measures (19). Therefore, it is recommended to enhance operational guidance and supervision for HCWs in isolation wards, regularly evaluate and provide feedback on the utilization and efficacy of PPE, and promptly identify and rectify any existing issues or deficiencies. By establishing an organic and unified personal protection culture of KAP in the clinic, the health and safety of frontline HCWs in the prevention and control of infectious diseases can be truly guaranteed.

### Analysis of factors affecting personal protection among HCWs in isolation wards

The analysis of factors affecting personal protection among HCWs in isolation wards of designated hospitals uncovers several significant influences. Notably, the frequency of trainings on protective equipment, years of experience, cumulative time spent working in isolation wards, and levels of anxiety and depression emerge as pivotal variables.

Our study observed a substantial positive correlation between the number of trainings attended and HCWs’ KAP regarding personal protection. By providing numerous training opportunities, the competence and confidence of HCWs in personal protection can be markedly enhanced (20). These training sessions often encompass both theoretical knowledge transfer and practical skill development, ensuring HCWs remain updated with the latest infection control guidelines and best practices. Consequently, this not only augments their knowledge but also refines their proficiency in practical applications, such as the proper donning and doffing of PPE. A Polish survey further underscores the necessity of educating medical staff on the appropriate use and selection of PPE (6). Our findings revealed that HCWs who participated in multiple trainings exhibited heightened levels of comprehensive knowledge and skills in managing infectious diseases, thereby bolstering their personal protection capabilities within isolation wards.

Additionally, the research indicates a significant positive correlation between the working years of medical personnel and their level of personal protection KAP. Healthcare practitioners with longer work experience generally demonstrate higher proficiency in personal protection, attributed to the extensive expertise accumulated over years of service in handling patients with infectious diseases (21). As healthcare professionals gain more experience, they develop a heightened awareness regarding hospital infection management, prompting them to implement personal protective measures more effectively to safeguard both their own and their patients’ safety.

The cumulative working hours of HCWs in isolation wards also positively correlate with their adherence to personal protective behaviors and their level of KAP. Prolonged exposure to the isolation ward environment allows HCWs to accumulate essential knowledge and practical experience in personal protection, enhancing their confidence and facilitating accurate execution, particularly in high-stress settings.

The presence of high anxiety levels among HCWs can result in a decline in their personal protection knowledge scores, indicating a significant impact on their knowledge dimension. Anxiety was linked to disruptions in learning and information processing, potentially reducing individuals’ ability to concentrate and acquire new knowledge effectively, as highlighted by Klassen’s self-efficacy theory (22). Cognitive disruptions such as distractibility, memory difficulties, and reduced information processing speed can render HCWs more susceptible to omissions or errors when adopting personal protective measures. Furthermore, anxiety significantly influences the personal protective practice dimensions of HCWs, with high levels negatively correlated with irregular personal protective behaviors. This may be attributed to decreased decision-making and behavioral execution abilities caused by anxiety, as suggested by LeBlanc’s study (23). Therefore, high anxiety levels may cause HCWs to feel nervous and make mistakes or omissions when applying personal protective measures, ultimately affecting their practice scores.

Similarly, the level of depression significantly influences the dimensions of personal protection attitude among HCWs. A depressive state is associated with negative attitudes toward personal protective measures, indicating that HCWs experiencing depression may develop unfavorable views toward their work and exhibit unsupportive behaviors regarding personal protection. This finding aligns with Li et al.’s (24) study, which revealed a strong correlation between depressive mood, mental health, and work-related attitudes among HCWs during the pandemic. Depressive moods were associated with feelings of exhaustion, helplessness, and disinterest in one’s job, and altered perceptions of personal protective measures. Such negative attitudes were linked to increased resistance toward work and lower adherence to personal protective measures, ultimately compromising their effectiveness in safeguarding HCWs against potential risks.

### Personal protection beyond COVID-19

Although the immediate threat posed by COVID-19 has diminished, the findings of this study extend beyond the current pandemic context. For instance, while some researchers have explored interventions such as traditional medicine during and post-pandemic (25), our study specifically investigated the KAP of HCWs toward PPE in COVID-19 isolation wards, emphasizing how employment tenure, training frequency, and mental health status (e.g., anxiety and depression levels) shape their adherence to protective measures. These insights highlight that robust KAP frameworks are essential not only for COVID-19 but also for managing other infectious diseases (26). The identified principles, such as the critical role of frequent PPE training and the need to address mental health challenges (e.g., anxiety reduction), can be adapted to diverse high-risk clinical settings, including units managing highly contagious pathogens (17, 27). Importantly, mitigating mental health barriers (e.g., through targeted support for anxiety and depression) may enhance HCWs’ compliance with PPE protocols, thereby strengthening infection control practices in future outbreaks. It is imperative to maintain high standards of training and mental health support for healthcare workers, ensuring they are equipped to handle emerging threats or resurgence of known diseases effectively (28). Further research is needed to explore how the insights gained from this study could inform policies and practices in non-COVID-19 related infectious disease wards.

While this study was conducted in isolation wards of designated hospitals in Shandong Province, China, the findings have broader implications for healthcare systems worldwide. The importance of PPE training, mental health support, and work experience in enhancing HCWs’ KAP is not limited to a specific region or country. Studies from other countries, such as the United States (29), South America (30), and Europe (31), have similarly highlighted the critical role of PPE training and psychological well-being in ensuring effective personal protection during pandemics.

## Conclusion

The results of this study indicate that the HCWs in the isolation wards of the designated COVID-19 treatment hospital demonstrated strong personal protection awareness and effective implementation of preventive measures, with favorable attitudes and practices. The study revealed associations between higher frequency of personal protective equipment training, lower anxiety levels and improved personal protection knowledge. Additionally, lower levels of depression were associated with more positive attitudes toward personal protective measures. HCWs with longer work experience and cumulative working time in isolation wards exhibited stronger adherence to personal protection regulations, which coincided with receiving mental health support, particularly interventions for anxiety.

However, the study has limitations: for instance, the use of convenience sampling and single-site data in this research may limit the generalizability of the findings. Additionally, the findings are specific to the isolation ward of a single designated hospital in Shandong Province, China, and thus may not represent the circumstances of HCWs in other hospitals or regions globally. The cross-sectional design also may restrict conclusions about long-term trends or causality. Self-reported data could be subject to social desirability bias. Future research should adopt broader sampling methods and longitudinal designs to validate these findings and enhance global applicability.

## Data Availability

The original contributions presented in the study are included in the article/Supplementary material, further inquiries can be directed to the corresponding author.
