# Supplementary material for: Current status and influencing factors of knowledge, attitude and practice of personal protection of healthcare workers in isolation wards of COVID-19 designated hospitals
Source: Front Public Health. 2025 May 14;13:1510015. doi: 10.3389/fpubh.2025.1510015 (PMC12116540; doi:10.3389/fpubh.2025.1510015)
Supplement: Supplementary file 1 [file Data_Sheet_1.docx]

**Appendix 1. Informed Consent**

Dear respondent,

Thank you for your willingness to participate in our questionnaire survey on the knowledge, attitude, and practice of personal protection among medical staff in COVID-19 isolation wards. This survey aims to gain insights into the knowledge, attitude, and practice of healthcare workers working in COVID-19 isolation wards regarding personal protection. The objective is to enhance safety awareness among healthcare workers and provide a basis for optimizing subsequent protective measures.

The following provides relevant information about this survey:

**Survey content:** Knowledge, attitude, and practice related to personal protection among healthcare workers in COVID-19 isolation wards.

**Confidentiality principle:** All information collected during this survey will be strictly confidential and used solely for academic research purposes without disclosing any personal details.

**Survey duration:** It is expected to take approximately 10-15 minutes.

**Participation method:** Please scan the QR code below or click the link provided to complete the online questionnaire.

We understand that your participation is crucial for this survey; therefore, we have implemented various measures to ensure the protection of your privacy and rights. If you have any questions or require assistance at any time, please do not hesitate to contact us using the following information: 0531-83347283.

Once again, thank you for your support and cooperation! We wish you success in your work and good health!

Please note that completing the online questionnaire will be considered as signing the informed consent. Next, please fill out the following questionnaire according to your general information, personal protection knowledge, attitude and practice status, and recent emotional state.

**Appendix 2. General Information Questionnaire**

1. Your gender:

○ Male

○ Female

1. Your age:

○ 18-25 years old

○ 26-30 years old

○ 31-40 years old

○ 41-50 years old

○ 51-60 years old

1. Your occupation:

○ Doctor

○ Nurse

○ Others

Others please note: such as cleaning, maintenance, security, etc.

1. Your professional title:

○ None or assistant title

○ Junior title

○ Intermediate title

○ Senior title

1. Your years of work in the medical industry:

○ Within 1 year

○ 1-3 years old

○ 4-6 years old

○ 7 to 10 years old

○ More than 10 years old

○ Others (Non-medical industry employees, such as cleaning, maintenance personnel, please select this option)

1. Your marital status:

○ Married

○ Single

1. Your children's status:

○ No children

○ Adult children (over 16 years old)

○ Minor children (less than 16 years old, with a capable second guardian to assist)

○ Minor children (less than 16 years old, without a capable second guardian to assist)

1. The cumulative number of days you have worked in the isolation ward:

○ 0-14 days

○ 15-30 days

○ 31-60 days

○ 61-100 days

○ More than 100 days

1. The status of your participation in protective equipment training:

○ No training

○ 1-2 times of training

○ 3-5 times of training

○ More than 5 times of training

**Appendix 3. KAP Questionnaire**

**Knowledge Dimension Questionnaire**

1. According to the “Diagnosis and Treatment Plan of COVID-19 Disease (Ninth Edition) ”, what are the main transmission routes of COVID-19 virus?

○ A. Vertical transmission and respiratory transmission.

○ B. Respiratory droplets and close contact transmission.

○ C. Close contact transmission and aerosol transmission.

○ D. Aerosol transmission and respiratory droplets transmission.

○ E. Unclear.

2. What is the incubation period after infection with Omicron variants?

○ A. 2-4 days.

○ B. 3-5 days.

○ C. 5-7 days.

○ D. 7-10 days.

○ E. unclear.

3. What should staff in the isolation ward wear when entering the contaminated area?

○ A. Primary protection.

○ B. Secondary protection.

○ C. Third-level protection.

○ D. Unclear.

1. What should staff in the isolation ward do when removing their protective masks?

○ A. Remove the upper and lower straps at the same time.

○ B. Remove the straps first and then remove the straps.

○ C. Remove the straps first and then remove the straps.

○ D. Unclear.

5. Which statement about hand hygiene is correct?

○ A. As long as there are gloves, there is no need to worry about microbial contamination to the hands.

○ B. Doctors should use quick-drying hand disinfectant to disinfect hands before physical examination of patients.

○ C. Hand hygiene is not required when entering or leaving the isolation ward and ICU and other key departments.

○ D. The six-step washing method must be done sequentially.

○ E. Unclear.

6. When wearing a protective mask, a tightness test should be performed on the protective mask. Which of the following statements is correct?

○ A. Press the nose clip with both hands and blow.

○ B. Cover the edge of the mask with both hands and exhale.

○ C. Drum the hands into a funnel shape, place them on the front of the mask, and exhale.

○ D. Drum the hands into a trumpet shape, place them on the front of the mask, and exhale.

○ E. Not clear.

7. When removing protective equipment in the first removal area, which of the following statements is incorrect?

○ A. After hand hygiene, take off the goggles or face screen first.

○ B. After hand hygiene, the gloves can hold the inside of the protective clothing.

○ C. When removing the protective clothing, the actions should be gentle.

○ D. When removing the protective clothing, they should be rolled down.

○ E. Not clear.

8. When removing protective equipment in the second removal area, which of the following statements is incorrect?

○ A. Take off the hat first and then take off the protective mask.

○ B. After removing the protective mask, put on the surgical mask as soon as possible.

○ C. When entering the second removal area, keep the ultraviolet lamp on.

○ D. When removing the hat, take it off quickly forward and downward to avoid rubbing the hair.

○ E. Not clear.

9. When in a contaminated area, regarding what should be done if protective clothing is damaged, which of the following statements is correct?

○ A. Spray the whole body of the protective clothing with 75% alcohol or quick-drying hand sanitizer.

○ B. Tear the protective clothing at the damaged place and put on another one.

○ C. Spray the damaged place with 75% alcohol or quick-drying hand disinfectant 5 times the diameter.

○ D. Spray the damaged place with 75% alcohol or quick-drying hand disinfectant 3 times the diameter.

○ E. Not clear.

10. In the contaminated area, regarding what to do if a protective mask falls off or becomes loose, which of the following statements is correct?

○ A. Without delaying the work, do not deal with it.

○ B. After cleaning hands, take off the mask in the contaminated area.

○ C. After cleaning hands, immediately cover the mask with hands and retreat to the first stripping area.

○ D. Put on another mask outside the loose mask.

○ E. Not clear.

**Attitude Dimension Questionnaire**

| Question | Very disagree | Disagree | Generally agree | Agree | Very agree |
| --- | --- | --- | --- | --- | --- |
| 1. Do you think it is important to conduct training and assessment on hospital infection knowledge and putting on and taking off protective equipment? | ○ | ○ | ○ | ○ | ○ |
| 2. Do you think it is important to be familiar with the brands and models of protective equipment commonly used in hospital? | ○ | ○ | ○ | ○ | ○ |
| 3. Do you think it is important to master necessary occupational protection knowledge to reduce occupational exposure hazards caused by COVID-19? | ○ | ○ | ○ | ○ | ○ |
| 4. Do you think it is necessary to learn emergency response procedures for occupational exposure in isolation wards? | ○ | ○ | ○ | ○ | ○ |
| 5. Do you think that repeatedly practicing putting on and taking off protective equipment and watching videos showing the details of putting on and taking off protective equipment will help you improve your skills and prevent occupational exposure? | ○ | ○ | ○ | ○ | ○ |
| 6. How effective do you think existing protective equipment is in protecting medical staff? | ○ | ○ | ○ | ○ | ○ |

**Practice Dimension Questionnaire**

| Question | Never | Occasionally | Sometimes | Often | Always |
| --- | --- | --- | --- | --- | --- |
| 1. Have you taken the initiative to repeatedly watch the video showing the process of putting on and taking off protective equipment and inquire about relevant prevention and control knowledge? | ○ | ○ | ○ | ○ | ○ |
| 2. Have you actively checked and studied COVID-19 prevention and control plans issued by the country? | ○ | ○ | ○ | ○ | ○ |
| 3. When taking off protective equipment, if you are not sure whether there is contamination, do you take the initiative to review the surveillance video to find out the reason? | ○ | ○ | ○ | ○ | ○ |
| 4. Have you taken the initiative to ask experienced colleagues, head nurses, and full-time hospital infection control personnel for advice on protective equipment? | ○ | ○ | ○ | ○ | ○ |
| 5. In the removal area, do you perform hand hygiene as required every time? | ○ | ○ | ○ | ○ | ○ |
| 6. Are you able to strictly implement the COVID-19 hospital infection prevention and control regulations in the isolation ward? | ○ | ○ | ○ | ○ | ○ |
| 1. When you take off your protective clothing, can you avoid contaminating the inner scrubs? | ○ | ○ | ○ | ○ | ○ |
| 8. In the removal area, do you perform hand hygiene for a sufficient length of time each time? | ○ | ○ | ○ | ○ | ○ |
| 9. When you wear a protective mask, do you perform an effective tightness test every time? | ○ | ○ | ○ | ○ | ○ |
| 10. When you take off your protective mask, can the vibration of the mask be controlled to a small range? | ○ | ○ | ○ | ○ | ○ |

**Appendix 4. Anxiety and Depression Questionnaire**

**Beck Anxiety Inventory (BAI)**

Instructions: Below is a list of common symptoms of anxiety. Indicate how much you have been bothered by that symptom during the past week, including today. Please carefully read each item in the list and make the corresponding choices.

|  | Not At All | Mildly but it  didn’t  bother me  much | Moderately -  it wasn’t  pleasant at  times | Severely – it  bothered  me a lot |
| --- | --- | --- | --- | --- |
| 1. Numbness or tingling | ○ | ○ | ○ | ○ |
| 2. Feeling hot | ○ | ○ | ○ | ○ |
| 3. Wobbliness in legs | ○ | ○ | ○ | ○ |
| 4. Unable to relax | ○ | ○ | ○ | ○ |
| 5. Fear of worst happening | ○ | ○ | ○ | ○ |
| 6. Dizzy or lightheaded | ○ | ○ | ○ | ○ |
| 7. Heart pounding/racing | ○ | ○ | ○ | ○ |
| 8. Unsteady | ○ | ○ | ○ | ○ |
| 9. Terrified or afraid | ○ | ○ | ○ | ○ |
| 10. Nervous | ○ | ○ | ○ | ○ |
| 11. Feeling of choking | ○ | ○ | ○ | ○ |
| 12. Hand trembling | ○ | ○ | ○ | ○ |
| 13. Shaky/unsteady | ○ | ○ | ○ | ○ |
| 14. Fear of losing control | ○ | ○ | ○ | ○ |
| 15. Difficulty in breathing | ○ | ○ | ○ | ○ |
| 16. Fear of dying | ○ | ○ | ○ | ○ |
| 17. Scared | ○ | ○ | ○ | ○ |
| 18. Indigestion | ○ | ○ | ○ | ○ |
| 19. Faint/lightheaded | ○ | ○ | ○ | ○ |
| 20. Face flushed | ○ | ○ | ○ | ○ |
| 21. Hot/cold sweats | ○ | ○ | ○ | ○ |

**Beck Depression Inventory (BDI)**

Instructions: This questionnaire is made up of a number of groups of items. Please read the items in each group carefully and then choose the statement in each group that best fits your current situation [last week, including today]. Please read all the statements in a group first and then choose.

1.

○ I do not feel sad.

○ I feel sad.

○ I am sad all the time and I can't snap out of it.

○ I am so sad and unhappy that I can't stand it.

2.

○ I am not particularly discouraged about the future.

○ I feel discouraged about the future.

○ I feel I have nothing to look forward to.

○ I feel the future is hopeless and that things cannot improve.

3.

○ I do not feel like a failure.

○ I feel I have failed more than the average person.

○ As I look back on my life, all I can see is a lot of failures.

○ I feel I am a complete failure as a person.

4.

○ I get as much satisfaction out of things as I used to.

○ I don't enjoy things the way I used to.

○ I don't get real satisfaction out of anything anymore.

○ I am dissatisfied or bored with everything.

5.

○ I don't feel particularly guilty.

○ I feel guilty a good part of the time.

○ I feel quite guilty most of the time.

○ I feel guilty all of the time.

6.

○ I don't feel I am being punished.

○ I feel I may be punished.

○ I expect to be punished.

○ I feel I am being punished.

7.

○ I don't feel disappointed in myself.

○ I am disappointed in myself.

○ I am disgusted with myself.

○ I hate myself.

8.

○ I don't feel I am any worse than anybody else.

○ I am critical of myself for my weaknesses or mistakes.

○ I blame myself all the time for my faults.

○ I blame myself for everything bad that happens.

9.

○ I don't have any thoughts of killing myself.

○ I have thoughts of killing myself, but I would not carry them out.

○ I would like to kill myself.

○ I would kill myself if I had the chance.

10.

○ I don't cry any more than usual.

○ I cry more now than I used to.

○ I cry all the time now.

○ I used to be able to cry, but now I can't cry even though I want to.

11.

○ I am no more irritated by things than I ever was.

○ I am slightly more irritated now than usual.

○ I am quite annoyed or irritated a good deal of the time.

○ I feel irritated all the time.

12.

○ I have not lost interest in other people.

○ I am less interested in other people than I used to be.

○ I have lost most of my interest in other people.

○ I have lost all of my interest in other people.

13.

○ I make decisions about as well as I ever could.

○ I put off making decisions more than I used to.

○ I have greater difficulty in making decisions more than I used to.

○ I can't make decisions at all anymore.

14.

○ I don't feel that I look any worse than I used to.

○1 I am worried that I am looking old or unattractive.

○ I feel there are permanent changes in my appearance that make me look

Unattractive.

○ I believe that I look ugly.

15.

○ I can work about as well as before.

○ It takes an extra effort to get started at doing something.

○ I have to push myself very hard to do anything.

○ I can't do any work at all.

16.

○ I can sleep as well as usual.

○ I don't sleep as well as I used to.

○ I wake up 1-2 hours earlier than usual and find it hard to get back to sleep.

○ I wake up several hours earlier than I used to and cannot get back to sleep.

17.

○ I don't get more tired than usual.

○ I get tired more easily than I used to.

○ I get tired from doing almost anything.

○ I am too tired to do anything.

18.

○ My appetite is no worse than usual.

○ My appetite is not as good as it used to be.

○ My appetite is much worse now.

○ I have no appetite at all anymore.

19.

○ I haven't lost much weight, if any, lately.

○ I have lost more than five pounds.

○ I have lost more than ten pounds.

○ I have lost more than fifteen pounds.

20.

○ I am no more worried about my health than usual.

○ I am worried about physical problems like aches, pains, upset stomach, or

constipation.

○ I am very worried about physical problems and it's hard to think of much else.

○ I am so worried about my physical problems that I cannot think of anything else.

21.

○ I have not noticed any recent change in my interest in sex.

○ I am less interested in sex than I used to be.

○ I have almost no interest in sex.

○ I have lost interest in sex completely.
